# Exposure to artificial lighting at night: from an ecological challenge to a risk factor for glucose dysmetabolism and gestational diabetes? Narrative review

**DOI:** 10.1080/07853890.2025.2477304

**Published:** 2025-03-11

**Authors:** Lina Zabuliene, Charalampos Milionis, Eftychia Koukkou, Ioannis Ilias

**Affiliations:** a Faculty of Medicine, Vilnius University, Vilnius, Lithuania; b Department of Endocrinology, Diabetes and Metabolism, Elena Venizelou General and Maternity Hospital, Athens, Greece; c Department of Endocrinology, Hippokration General Hospital, Athens, Greece

**Keywords:** Light pollution, global health, melatonin, gestational diabetes mellitus, fetal development, macrosomia

## Abstract

Introduction: Artificial lighting at night (ALAN) leads to pervasive light pollution, affecting ecosystems and human health globally. Satellite assessments reveal widespread nocturnal illumination worldwide and research indicates adverse health effects. Environmental light pollution disrupts natural cycles, affecting the behavior and reproduction of various organisms. Aim/Method: In this narrative review we aimed to present research on the effects of ALAN on glucose metabolism and diabetes and hone on its recently reported association with gestational diabetes (GDM). Results: Conflicting data exist on the effects of melatonin’s administration vis-à-vis glycemia, with some studies suggesting beneficial outcomes for patients with type 2 diabetes mellitus and insomnia. Ambient light influences plasma glucose, with bright light increasing both fasting and postprandial glucose levels. Perinatal light exposure is linked to later-life health risks and prenatal exposure to ALAN is linked to fetal macrosomia. Analyzing European ALAN data in conjunction with epidemiological records for GDM reveals a notable probable association. Additionally, recent research from China (one case-control and two cohort studies) has shown that exposure to ALAN during pregnancy significantly increases the risk of GDM. Discussion/Conclusion: Despite progress, interdisciplinary research is needed to understand the impact of light pollution on health, especially regarding disrupted light-dark cycles and physiological functions relevant to conditions like GDM. At present, the simplest advice for all people and particularly for women who anticipate pregnancy, or for pregnant women, is to ensure a totally dark environment during sleep time.

## 1. Introduction - Artificial lighting at night

Pathologic exposure to artificial lighting at night (ALAN) refers to excessive or inappropriate illumination during nighttime hours, disrupting normal physiological and behavioral processes. Such exposure interferes with circadian rhythms, triggering adverse health effects, including hormonal imbalances and metabolic disturbances [1–5]. A significant contributor is high-intensity light, especially short-wavelength blue light (460–480 nm), which suppresses melatonin by activating intrinsically photosensitive retinal ganglion cells (ipRGCs). These disruptions are exacerbated by nighttime exposure, when the body relies on darkness for physiological restoration.

Before the advent of ALAN, human sleep patterns were predominantly biphasic, characterized by a wake period around midnight [6,7]. Adaptations to extreme latitudes, such as in the Arctic and Antarctic, highlight the challenges posed by prolonged periods of darkness or daylight. Seasonal variations in light availability disrupt circadian rhythms, causing insomnia, delayed sleep-wake cycles, and mood disturbances [8–10]. While indigenous populations, such as the Sámi, demonstrate adaptive strategies, including more flexible sleep regulation and behavioral practices, which mitigate these effects [11], this may not be the case for transient/temporary inhabitants of extreme latitudes. Seasonal affective disorder (SAD), characterized by depressed mood, hypersomnia, and overeating, emerges during prolonged winter darkness. Elevated melatonin levels during the polar night exacerbate fatigue and impair daily functioning [8,9]. Exposure to enriched blue or bluish-green light, such as wavelengths around 505 nm, has shown promise in improving mood and sleep quality, as evidenced by studies in Arctic military personnel and Antarctic research stations [12–14]. Lifestyle interventions, including consistent physical activity, have also demonstrated efficacy in reducing afternoon melatonin levels and promoting wakefulness [15].

The widespread availability of ALAN has shifted human sleep patterns from biphasic to monophasic, amplifying its health impacts, especially in contexts like shift work, urban light pollution, and late-night screen use. Normally, the cortisol secretory profile lags behind that of melatonin’s secretion, but it is not affected by factors such as sleep deprivation [16]. Chronic exposure to ALAN disrupts circadian rhythms, suppresses melatonin, and dysregulates cortisol secretion. Such disruptions contribute to increased risks of obesity, diabetes, cardiovascular disease, mood disorders, cognitive dysfunction, and even certain cancers [17–20]. The aim of this narrative review was to summarize and assess the effects of ALAN on glucose metabolism/dysmetabolism, diabetes and the recently reported association of ALAN with gestational diabetes (GDM).

## 2. Light pollution: a contemporary challenge

Light pollution, defined as the excessive and inappropriate use of ALAN, significantly impacts human health, ecosystems, and the environment. Initially noted by astronomers for its interference with celestial observations, its broader implications have since been recognized, encompassing disruptions to biological processes and ecological systems [21]. Urbanization and population growth have intensified light pollution, with global assessments revealing its pervasive nature [22,23].

Satellite technologies have been instrumental in quantifying ALAN’s global footprint, but limitations persist, such as the inability to measure indoor exposure or capture all wavelengths of ALAN. Ground-based validation complements satellite data, addressing gaps in resolution and accuracy [24,25]. These assessments underscore the growing recognition of light pollution as an environmental pollutant with profound health implications.

As stated above, aberrant light exposure disrupts the biological clock, governed by light-dark cycles, and alters melatonin production, the key hormone regulating sleep and other physiological processes. This dysregulation is linked to various health issues, including increased risks of depression, sleep disturbances, and cancer [26–28]. With regards to cancer, experimental evidence is quite strong: mice exposed to excess light and irregular light/dark cycles may develop mammary gland cancer [29,30]. As for humans, there is accumulating evidence of a link between circadian rhythm disruption (associated with shift work and exposure to ALAN). In fact, the International Agency for Research on Cancer (IARC) classifies shiftwork, involving circadian disruption as being probably carcinogenic [31].

The definition of excess exposure to ALAN could be problematic. Yet, there is a consensus from an international workshop [32] and there are guidelines on what constitutes accepted levels for nighttime indoor ALAN levels, particularly for the sleep environment [33]. The levels of outdoor ALAN, in the studies originating from China are very high, in the order of 43x10^6^ lux [34]. Taking into account the fact that people spend time outside for commuting, work or recreation and that light is “pervasive”, indoor lighting is heavily influenced by outdoor lighting.

Emerging evidence also highlights a combined impact of ALAN and air pollution on health, as demonstrated by studies in China, where cardiovascular risks are exacerbated by high outdoor ALAN levels [34]. Indoor lighting exposure, influenced by outdoor sources, often exceeds recommended thresholds for sleep environments, further compounding health risks [33]. For instance, LED screens emit approximately 40 lux, far exceeding the 1-lux guideline for nighttime lighting.

Addressing light pollution requires a multifaceted approach, including international guidelines on ALAN exposure, improved urban planning, and public awareness campaigns. These efforts aim to mitigate the adverse effects of ALAN on health and the environment, emphasizing the importance of preserving natural darkness for human well-being and ecological balance [35–37].

## 3. Melatonin and the homeostasis of glucose

Melatonin, through its action on MT1 and MT2 receptors in pancreatic beta cells, mainly prevents insulin secretion by beta cells as well as reducing cyclic AMP and cyclic GMP, improving recovery and cell survival [38]. It thus creates a state necessary for insulin sensitivity during the day [39]. Peak plasma melatonin at late night (02:00-04:00 h) ranges from 100 to 200 pg/mL [40]; melatonin levels range from 10 to 30 pg/mL during the day [41]. Illumination from 200 to 600 lux inhibits melatonin secretion [42]. Based on these data, reduced melatonin secretion is a risk factor for the development of insulin resistance and exacerbation of type 2 Diabetes Mellitus (DM2) [43,44]. While melatonin’s potential effects on glucose management and DM2 have garnered attention, the available relevant data are conflicting. Studies suggest both ameliorative and exacerbating effects, with some linking melatonin to improved glucose tolerance and glycated hemoglobin (HbA1c) levels in DM2 patients with insomnia, and others suggesting potential exacerbation, particularly in those with a melatonin receptor (MTNR1B; rs10830963 SNP (C/G)) risk variant [45]. As stated above, melatonin has been implicated in the modulation of insulin release within the pancreatic islets. However, it should be stressed that the timing of meals also plays an important role in glucose homeostasis, as in the case of night workers (who present with increased melatonin concentration) or melatonin users who take their meals during times of increased melatonin bioavailability. This was also confirmed by a randomized study of 845 subjects (71% women, mean ± SD age: 38 ± 14 years; mean ± SD BMI: 25.67 ± 4.69 kg/m^2^; all the subjects were healthy, of European descent and not taking medications, including melatonin) who consumed late-night meals [46]. More in detail, a glucose tolerance test was performed 4 h (first group) or 1 h before night sleep (second group). Serum melatonin was significantly elevated in the second group, where increased glucose and decreased insulin activity were observed. The authors concluded that avoiding late night meals could improve insulin sensitivity and the glycemic profile in humans. In the very early morning hours, melatonin concentrations are elevated, and researchers –albeit based on small-scale studies – point to the interest for future research investigating whether postponing breakfast, following an early morning awakening, could serve as a potential therapeutic strategy for enhancing metabolic health [47].

## 4. Artificial light at night and glucose metabolism/diabetes

Epidemiological studies, including those focused on individuals involved in shift work and night work, have underscored the association between sustained night work and an elevated incidence of various diseases, including cancer, diabetes, cardiovascular risks, obesity, mood disorders, and age-related macular degeneration [28,48]. ALAN has emerged as a potential disruptor of the circadian timing system, with implications for various physiological processes and disease risk [49].

A comprehensive understanding of the impact of ALAN on metabolic parameters reveals its pervasive influence on glucose and lipid metabolism. Experimental studies have elucidated the role of ALAN, especially its effect on the biological clock in the suprachiasmatic nucleus of the hypothalamus, and its impact on various physiological processes, including locomotor activity, food intake, the sleep/wake cycle, body temperature, melatonin, glucocorticoids, glucose, and lipid metabolism [50]. Importantly, evidence points to the impact of ALAN on glucose homeostasis in humans, underscoring its association with the increasing incidence of obesity, overweight, and atherosclerosis [51]. The precise mechanisms remain under speculation. These mechanisms may include perturbations in wakefulness patterns, alterations in dietary behaviors, and potential modifications in the effects of reduced melatonin on insulin function [52–57].

Furthermore, acute effects of daytime, indoor, ambient light intensity on plasma glucose (and lipids) have been investigated (excluding circadian rhythm disruption) [58]: eight healthy, non-obese, young (< 25 years) men, without DM2 and eight overweight-obese, middle-aged (> 50 years) men, with DM2 on metformin, were studied. In a crossover fashion, they were exposed to either dim 910 lux) or intense (4000 lux) indoor light from 07:30 to 13:30, with a week’s interval. Standardized meals were given at 08:30 and several blood samples were obtained pre-and postprandially. In the healthy young men, conditions of bright versus dim indoor light increased fasting and postprandial triglycerides. In the subjects with DM2, intense indoor light was associated with increased fasting and postprandial glucose and postprandial triglycerides, compared to dim light. These results emphasize the need for further research into the implications of the acute metabolic effects of light for the prevention and diagnosis of hyperglycemia and dyslipidemia. Additionally, perinatal light exposure has been implicated in imprinting the circadian timing system, thereby influencing later-life physiology and disease risk. A cross-sectional investigation, using the UK Biobank data, revealed associations between perinatal photoperiod characteristics and the prevalence of DM2, emphasizing the importance of considering individual time-of-year of birth and corresponding latitude in understanding these relationships[59]. Notably, the study identified different effects based on the photoperiod experienced during the perinatal period, underscoring the potential non-linear dose-response to photoperiods or the confounding role of artificial light in these associations. A recent study in China aimed to investigate the associations between chronic exposure to outdoor LAN and markers of glucose homeostasis, as well as DM2 prevalence [60]. The study, conducted as part of the China Noncommunicable Disease Surveillance Study, involved 98,658 participants aged ≥ 18 years, with DM2, defined according to ADA criteria. Outdoor ALAN exposure was estimated from satellite data, revealing positive exposure-response associations between ALAN levels and HbA1c, fasting and 2-hour glucose concentrations, and HOMA-IR, while showing a negative monotonic association with HOMA-B; the authors do not provide p values but their reported 95% confidence intervals (CI) of prevalence rates suggest statistical significance. Notably, the highest quintile of ALAN exposure (median 69.1 mW/cm^2^/sr) was significantly associated with an increased prevalence of DM2 (prevalence ratio 1.28, 95% CI: 1.03 to 1.60) compared to the prevalence of lowest ALAN exposure quintile (median 1.0 mW/cm^2^/sr), emphasizing the potential role of outdoor ALAN as a novel risk factor for DM2.

Understanding the broader context, research over the past 60 years has highlighted a significant rise in metabolic diseases, particularly DM2, coinciding with the increasing prevalence of ALAN. This shift has led to alterations in behavioral cycles, sleep deprivation, and circadian misalignment, all of which have been implicated in the pathogenesis of diabetes. Although the exact mechanisms are not fully elucidated, experimental findings suggest a direct influence of sleep deprivation and circadian misalignment on the development of metabolic dysfunction and insulin resistance [61,62]. However, it is very difficult to pinpoint the metabolic effects of exposure to ALAN from concomitant and associated alterations in food intake and physical activity. We have to bear in mind that such alterations were even noted by the IARC when assessing the tumorigenic potential of shiftwork/circadian disruption (with the unavoidable exposure to ALAN). For now, the IARC terms shiftwork/circadian disruption as probably carcinogenic (category 2 A) [31]; other explanations for carcinogenesis, such as changes in nutrition or physical activity, cannot be ruled out – the same rationale should also apply for metabolic dysfunction.

## 5. Artificial light at night and gestational diabetes

The medical literature currently lacks direct and focused data on the relationship between GDM and ALAN. However, recent research from China has suggested a potential association, linking increased outdoor ALN exposure during pregnancy to higher fetal weight and an elevated risk of macrosomia, particularly in female fetuses [63] as well as a higher risk of preterm birth (PTB) [64]; these characteristics/outcomes are commonly observed in GDM [65,66].

More in detail, one study included data from 6,210 mother-child pairs, spanning the period from 2015 to 2021, and satellite imaging was used to measure the levels of outdoor ALAN during pregnancy. Fetal parameters, including biparietal diameter, head circumference, abdominal circumference (AC), and femur length, were measured using ultrasonography before delivery. Anthropometric data at birth, such as birth length, birth weight, and indicators like macrosomia, low birth weight, small for gestational age, and large for gestational age, were collected. In this study, 25% of the women had pregnancy complications (described by the authors as having gestational hypertensive disease and/or gestational diabetes) while 9% of their fetuses/neonates had macrosomia (11% in males and 7% in females). After accounting for confounding factors, the researchers found that increased outdoor ALAN during pregnancy was associated with higher AC percentiles and an elevated risk of macrosomia. Furthermore, when the data was stratified by sex, this association was particularly pronounced in female fetuses (an increase in outdoor ALAN of 14.87 nW/cm2/sr during pregnancy was associated with an odds ratio [OR] of 1.27 (95% CI: 1.06 to 1.53) of macrosomia in females) [63]. The authors concluded from the findings of the study that heightened exposure to outdoor ALAN during pregnancy is linked to larger fetal AC and an increased risk of macrosomia, especially in female fetuses. In the seminal Hyperglycemia and Adverse Pregnancy Outcome (HAPO), all levels of increased maternal blood glucose levels (even mild elevations, not reaching the threshold for GDM), were associated with an increased risk of adverse outcomes in pregnancy; macrosomia (affecting approximately 7%–15% of neonates) is considered to be such an outcome [67]. In another retrospective case–control study, the authors investigated the impact of outdoor ALAN exposure on PTB risk among 2,850 pregnant women in Beijing, China [64]. ALAN levels, estimated through remote sensing satellite data, were higher in the PTB group during the first and second trimesters. A negative association was observed between ALAN exposure and gestation days in both trimesters. ALAN emerged as a risk factor for PTB during the first (OR = 1.032, 95%CI: 1.025–1.040, *p* < 0.001) and second trimesters (OR = 1.018, 95%CI: 1.011–1.025, *p* < 0.001) of pregnancy [64]. Thus ORs of differences in the order of 1%–3% are reported in this study versus premature birth (PTB) - such differences, regardless of statistical significance, are not of clinical significance. Also from China, in a prospective population-based cohort study comprising 105,528 live singleton births from 2018 to 2020, maternal GDM was found to elevate the risk of PTB in both nulliparous (Adjusted Odds Ratio [ORadj] = 1.28, 95% Confidence Interval [CI]: 1.14–1.45) and multiparous (ORadj = 1.26, 95%CI: 1.14–1.40) mothers [66].

Additionally, there are indirect indications of an ALAN/GDM relationship. An examination of ALAN data in Europe against their corresponding epidemiological data for GDM, already available in published research works [68,69]- at least for data from Southern Europe–shows that the higher the exposure of the population to ALAN the higher the prevalence of GDM is (albeit such data are coarse and conclusions based on them are risky) [68,69] (See also Supplemental Figure 1a and b). Light pollution is a prevalent issue in Europe, exhibiting varying levels of intensity across different regions. While both northern and southern European countries are affected by light pollution, it may tend to be more severe in southern Europe. In certain regions of southern Europe, the population density is high, leading to an increased requirement for artificial light sources. Additionally, the warmer climate and sociocultural traditions in southern Europe promote outdoor activities at night, contributing to higher demand for lighting (and subsequent exposure to it). Further studies are necessary to validate these findings and elucidate the underlying mechanisms. ALAN data alongside epidemiological records pertaining to GDM could offer invaluable insights into this potential relationship.

Congruous to the above, three recent studies, all from China, have investigated the impact of exposure to ALAN on GDM. A case-control study involving 5,720 participants (1430 with GDM) found that higher ALAN exposure during pregnancy significantly increased GDM risk, with odds ratios (ORs) of 1.39 (95% CI: 1.20–1.63) and 1.70 (95% CI: 1.44–2.00) for the first trimester, and 1.70 (95% CI: 1.45–1.98) and 2.08 (95% CI: 1.77–2.44) for the second trimester, indicating a nonlinear relationship [70]. A significant nonlinear association (by means of restricted cubic spline analysis) was reported in the second trimester of pregnancy for ALAN > 4.235 nW/cm2/sr; the OR for GDM increased sharply from 1 to 2.5 (at 35–40 nW/cm2/sr) and then showed a very subtle increase for higher ALAN levels (p: 0.001). Similarly, a prospective cohort study from Sichuan, China, which included 9,120 participants(1484 with GDM), reported that higher ALAN levels were associated with increased GDM risk, with an OR of 1.253 (95% CI: 1.157–1.356) for the first two trimesters combined. The study also identified a nonlinear threshold effect at ALAN: 17.9 nW/cm^2^/sr and a plateau at ALAN: 40.7 nW/cm^2^/sr [71], in a manner analogous to the previous case-control study [70] and with a spline in the diagrams of OR for GDM versus level of exposure to ALAN at approximately the same level of ALAN (*p* < 0.003) with the preceding study. Another cohort study of 6,730 pregnant women in Hefei, China, found that first-trimester ALAN exposure was linked to a higher fasting plasma glucose of 0.02 mmol/L (95% CI: 0.00-0.03, p: 0.024), higher insulin levels of 0.42 μU/mL (95% CI: 0.30–0.54, *p* < 0.001), and of higher insulin resistance (HOMA-IR) by 0.09 (95% CI: 0.07–0.12, *p* < 0.001), particularly among those who conceived in summer and autumn [72]. This study also noted a spline in the graphs of (numerical) differences in FPG, insulin levels and HOMA-IR versus ALAN levels; differences also increased sharply up to approximately ALAN exposure at 40 nW/cm2/sr (*p* < 0.05) and then showed a plateau - of note the reported differences may be statistically significant but are too small to be of clinical significance. No differences in ORs are reported by the authors. The authors of these three studies conclude that exposure to ALAN during pregnancy may be a significant, modifiable risk factor for GDM.

## 6. Caveats and limitations regarding studies of the effect of ALAN on GDM

There are several caveats that should be taken into consideration, when examining the effects of ALAN on GDM. The first concerns the quality of epidemiological data on GDM. There could be concerns about it, and some studies, in the past, had yielded a very high prevalence (indicating that one-third of pregnancies are hampered by GDM, which may have been unrealistic [73]). Another caveat lies with the nature of ALAN per se, since there is a global shift towards ALAN with light emitting diodes’ (LED) sources, both indoors and outdoors [74]. LED lighting has a preponderant component of blue light (at a 100–500 nm wavelength). This LED lighting may have profound repercussions on animal and human circadian biology, probably, but not exclusively, due to a strong effect on the production of melatonin [75]. A third caveat is that there are also studies with indirectly divergent results [76], when compared to research work from China, regarding birth weight. In the United States, and more precisely in the state of New Jersey and over 5 years, researchers explored the impact of light pollution on fetal health by directly measuring skyglow (the artificial brightness of the night sky). Their study, in over 60000 subjects, revealed that light pollution was associated with decreased birth weight (mainly for male neonates), shortened gestational age, and increased incidence of preterm births. A fourth caveat concerns the magnitude of reported differences vis-à-vis levels of exposure to ALAN in some parameters, such as fasting glucose in pregnancy. Researchers have reported differences which are statistically significant, but, for now, are so small, that they are not of any meaningful clinical significance. A fifth caveat concerns the origin of most relevant studies. It seems that all of the negative effects of light exposure come from data acquired in China, which has its own culture, societal and health related behavior patterns, which may be different from those of Western countries. Finally, a sixth caveat concerns the type of relevant studies. It is important to note that the observational nature of the research precludes definitive conclusions about causation, and further research is needed to establish a causal relationship between ALAN and GDM.

## 7. Areas of future research

Despite the expanding body of research, there remains a need for interdisciplinary efforts to elucidate the ecological and physiological impacts of light pollution and exposure to ALAN. Future research should focus on longitudinal studies to delineate the long-term impact of ALAN on the development of GDM, thereby providing a more comprehensive understanding of how ALAN influences GDM over extended periods. Additionally, research still leaves unresolved the precise dose-response relationship between exposure to ALAN - its intensity and duration - and the risk of developing GDM, necessitating further investigation to clarify this relationship and establish specific exposure thresholds. While significant progress has been made in understanding the potential effects of exposure to ALAN on human health, additional research is warranted, particularly regarding the influence of dark periods on physiological functions relevant to diabetes, such as GDM.

## 8. Conclusion

Emerging evidence suggests a potential link between disrupted light-dark cycles, exposure to ALAN and altered glucose homeostasis in women at risk for or affected by GDM.

A more thorough exploration of the interplay between ALAN exposure, circadian rhythm disruption, and the pathogenesis of GDM seems imperative for conceiving, formulating and implementing effective preventive and therapeutic strategies. Research results presented in this review (along with future research) may have potential clinical implications: they could have repercussions in healthcare infrastructure and prompt possible modifications in the design of maternity wards. Finally, they could guide recommendations for home lighting during pregnancy; at present, the simplest advice for all people, and particularly for women who anticipate pregnancy or for pregnant women, is to ensure a totally dark environment during sleep time. Thus, the mitigation of exposure to ALAN may potentially reduce the risk of GDM among pregnant women.

## Supplementary Material

Suppl fig 1a and 1b 600 dpi.jpg

## Data Availability

Data sharing is not applicable to this article as no new data were created or analyzed in this study.

## Note

For illuminance (luminous flux per unit area), 1 watt/centimeter^2^/sr (W/cm^2^/sr) = 6830000.00000002 lux = 6830000.00000002 lumen/m^2^; for luminous intensity, 1 candela (cd) = 1 lumen/sr.
