# Supplementary figures and images for: Exposure to artificial lighting at night: from an ecological challenge to a risk factor for glucose dysmetabolism and gestational diabetes? Narrative review
Source: Ann Med. 2025 Mar 11;57(1):2477304. doi: 10.1080/07853890.2025.2477304 (PMC11899256; doi:10.1080/07853890.2025.2477304)

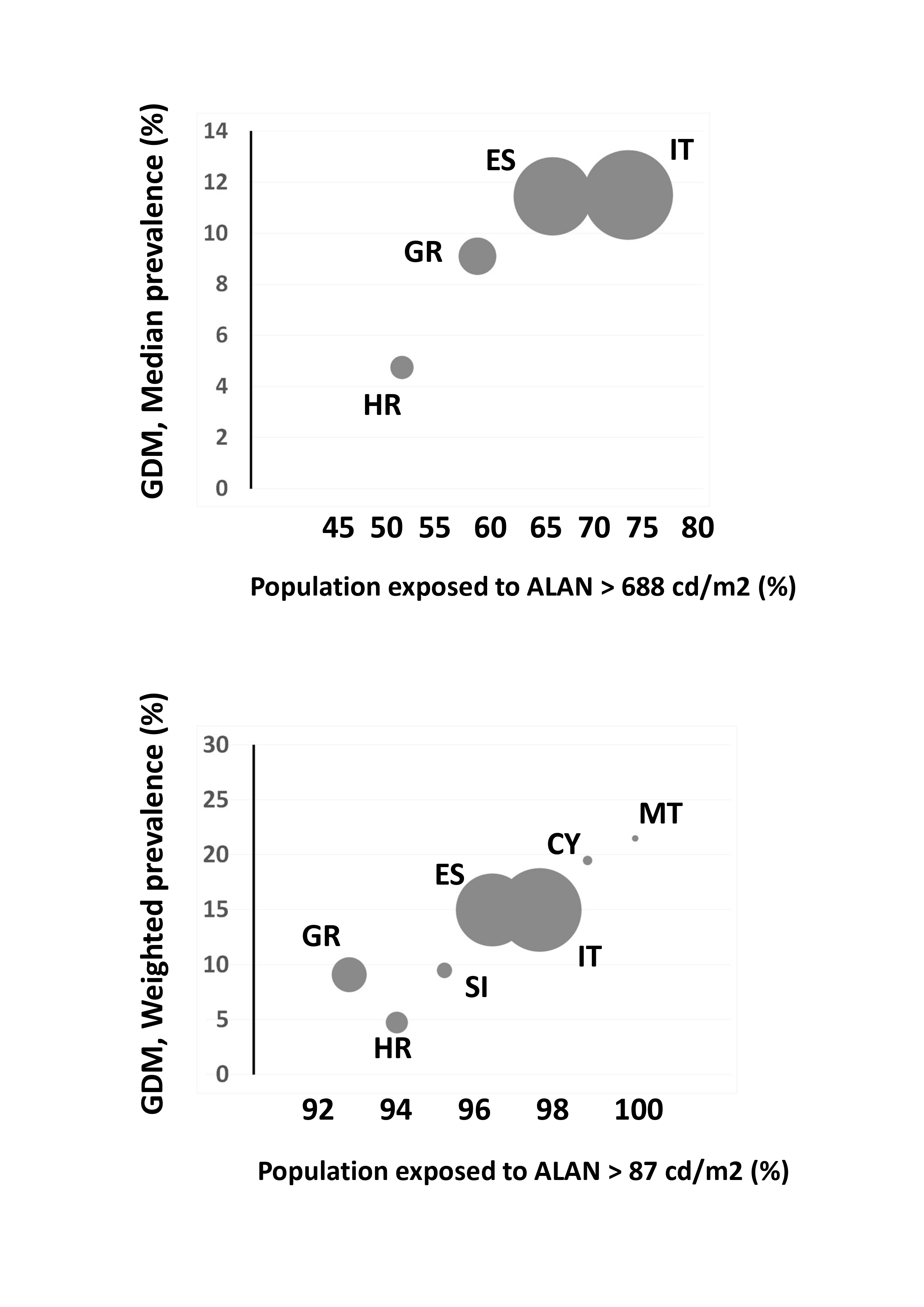

Supplement: Suppl fig 1a and 1b 600 dpi.jpg [file IANN_A_2477304_SM0758.jpg]
